# Supplementary material for: Temporal whole field sawtooth flicker without a spatial component elicits a myopic shift following optical defocus irrespective of waveform direction in chicks
Source: PeerJ. 2019 Jan 23;7:e6277. doi: 10.7717/peerj.6277 (PMC6347968; doi:10.7717/peerj.6277)

Part No.	AL-513W3C-A	Diff No.
5 mm	Round	Type : LED Lamps

Package Dimension：

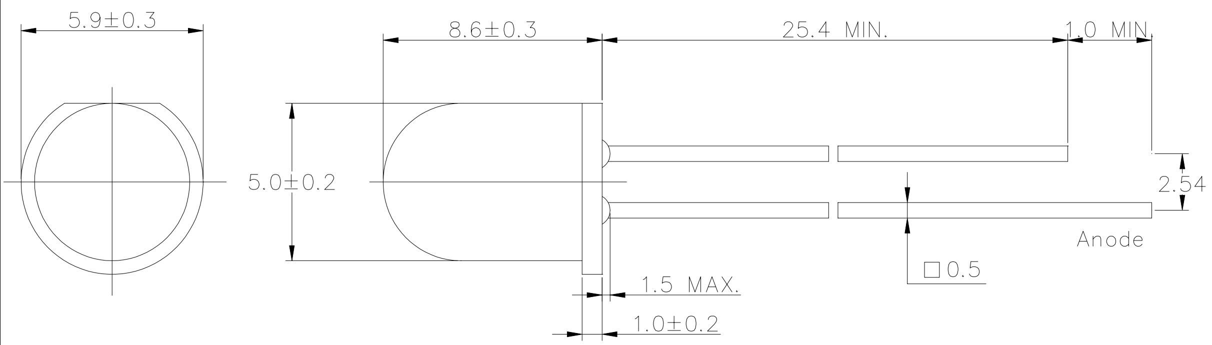

Notes:
1. All dimensions are in millimeter.
2.An epoxy meniscus may extend about.
  1. 5mm(0.059") down to the lead
3.Tolerances unless Dimension ±0.25mm

■ Features：

- Choice of various viewing angles.
- Available on Tape and Reel.
- Reliable and robust.

■ Descriptions：

- The series is specially designed for application requiring higher brightness.
- The LED lamps are available with different color, intensities, epoxy colors etc.

■ Applications：

- TV set
- Monitor
- Telephone

Part No. AL-513W3C-A Diff No.

5 mm Round Type : LED Lamps

PART NO.	Chip		Lens Color
	Material	Emitted Color	
AL-513W3C-A	InGaN	White	Water Clear

■ Absolute Maximum Ratings at Ta=25℃

Parameter	Symbol	Rating	Unit
Forward Current	I_F	30	mA
Operating Temperature	Topr	-40 to +85	℃
Storage Temperature	Tstg	-40 to +85	℃
Soldering Temperature	Tsol	260 ±5	℃
Electrostatic Discharge	ESD	1000	V
Power Dissipation	P_D	120	mW
Peak Forward Current (Duty 1/10@1KHz)	I_F (Peak)	100	mA
Reverse Voltage	V_R	5	V

■ Electronic Optical Characteristics：

Parameter	Symbol	Min.	Typ.	Max.	Unit	Condition
Luminous Intensity	I_V	5800	7000	／	mcd	I_F=20mA
Viewing Angle	2θ1/2	／	30	／	deg	I_F=20mA
Chromaticity Coordinates	X	／	0.31	／	／	I_F=20mA
	Y	／	0.32	／	／	I_F=20mA
Forward Voltage	V_F	／	3.2	3.5	V	I_F=20mA
Reverse Current	I_R	／	／	50	μA	V_R=5V

Part No.	AL-513W3C-A	Diff No.
5 mm	Round	Type ：LED Lamps

■ Reliability test items and conditions：

NO	Item	Test Conditions	Test Hours/Cycle	Sample Size	Ac/Re
1	Solder Heat	TEMP：260℃ ± 5℃	5 SEC	76 PCS	0/1
2	Temperature Cycle	H：+85℃ 30min<br>∫ 5min<br>L：-55℃ 30min	50 CYCLES	76 PCS	0/1
3	Thermal Shock	H：+100℃ 5min<br>∫ 10set<br>L：-10℃ 5min	50 CYCLES	76 PCS	0/1
4	High Temperature Storage	TEMP：100℃	1000 HRS	76 PCS	0/1
5	Low Temperature Storage	TEMP：-55℃	1000 HRS	76 PCS	0/1
6	DC Operating Life	TEMP：25℃<br>I_F=20mA	1000 HRS	76 PCS	0/1
7	High Temperature / High Humidity	85℃ / 85%RH	1000 HRS	76 PCS	0/1

Part No.	AL-513W3C-A	Diff No.
5 mm	Round	Type : LED Lamps

■ Color Ranks

	Rank a			
x	0.280	0.264	0.283	0.296
y	0.248	0.267	0.305	0.276

Approximate Color Temperature : 6500-10000K

	Rank b1			
x	0.287	0.283	0.330	0.330
y	0.295	0.305	0.360	0.339

Approximate Color Temperature : 5500-6500K

	Rank b2			
x	0.296	0.287	0.330	0.330
y	0.276	0.295	0.339	0.318

Approximate Color Temperature : 5500-6500K

	Rank C			
x	0.330	0.330	0.361	0.356
y	0.318	0.360	0.385	0.351

Approximate Color Temperature : 4500-5500K

■ CIE Chromatacity Diagram

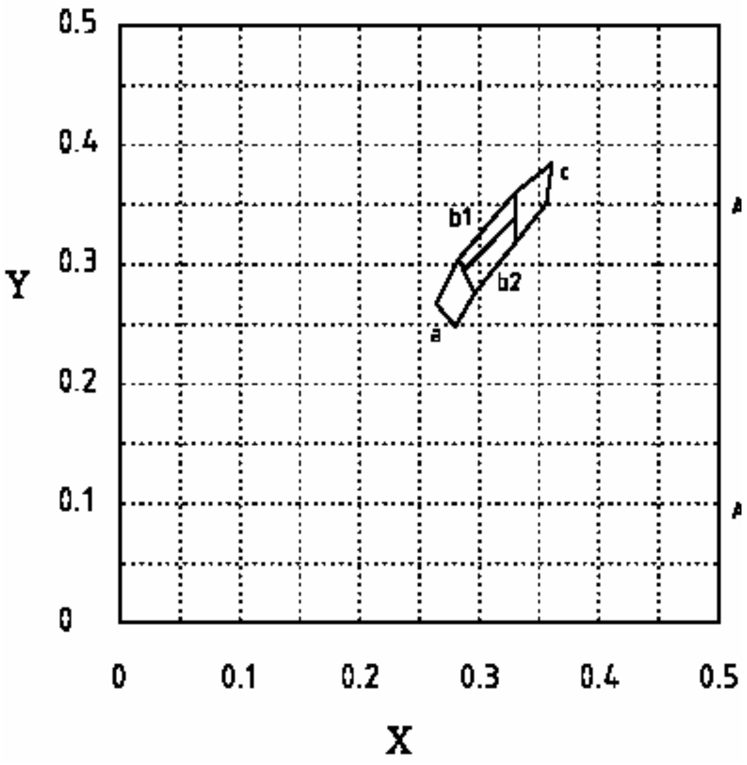

Part No.	AL-513W3C-A	Diff No.
5 mm	Round	Type : LED Lamps

◆ **TYPICAL ELECTRICAL-OPTICAL CHARACTERISTICS CURVES**

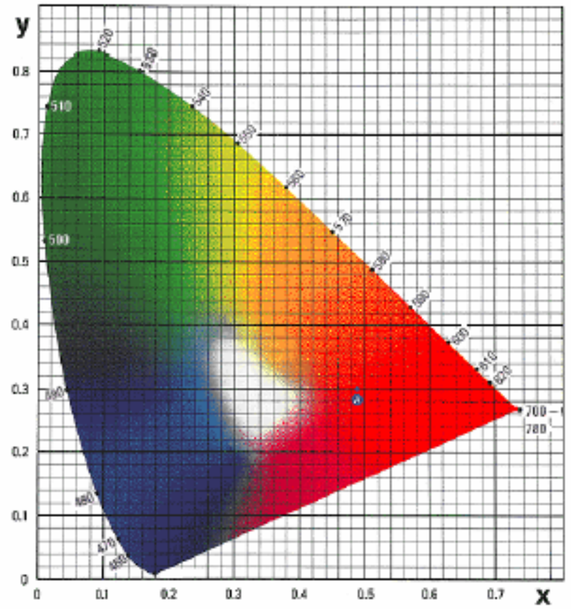

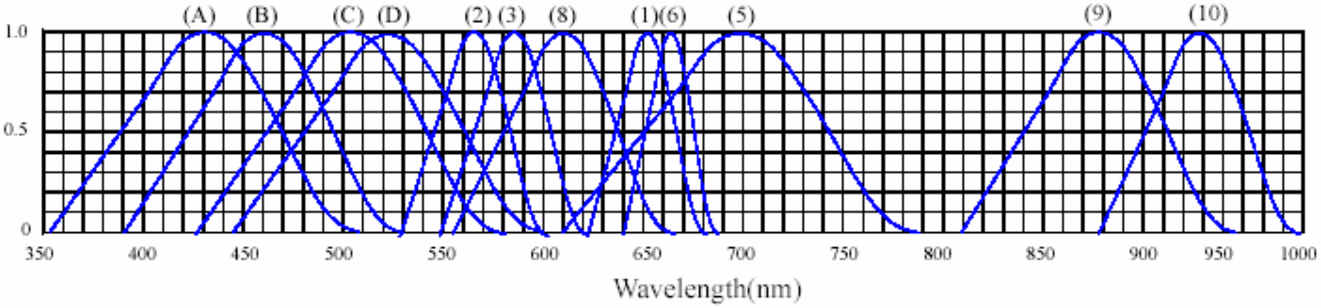

RELATIVE INTENSITY VS. WAVELENGTH(λp)

(1) GaAsP/GaAs 655nm/Red
(2) GaP 568nm/ Yellow Green
(3) GaAsP/GaP 585nm/Yellow
(4) GaAsP/GaP 635nm/Orange & Hi-Eff Red
(5) GaP 700nm/Bright Red
(6) GaAlAs/GaAs 660nm/Super Red
(8) GaAsP/GaP 610nm/Super Red

(9)- GaAlAs 880nm
(10)-GaAs/GaAs&GaAlAs/GaAs 940nm
(A)- GaN 430nm/Blue
(B)- InGaN 470nm/Blue
(C)- InGaN 502nm/Ultra Green
(D)- InGaN 523nm/Ultra Green

Part No.	AL-513W3C-A	Diff No.
5 mm	Round	Type : LED Lamps

◆ **CHARACTERISTICS DIAGRAMS**

FORWARD CURRENT VS. FORWARD VOLTAGE

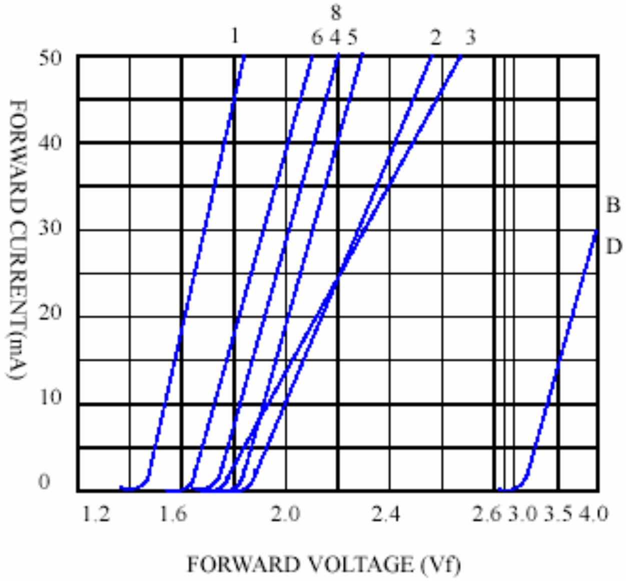

RELATIVE LUMINOUS INTENSITY VS. FORWARD CURRENT

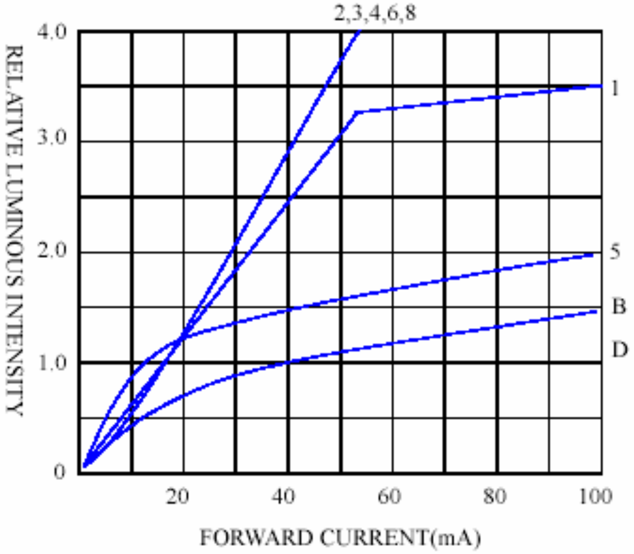

FORWARD CURRENT VS. AMBIENT TEMPERATURE

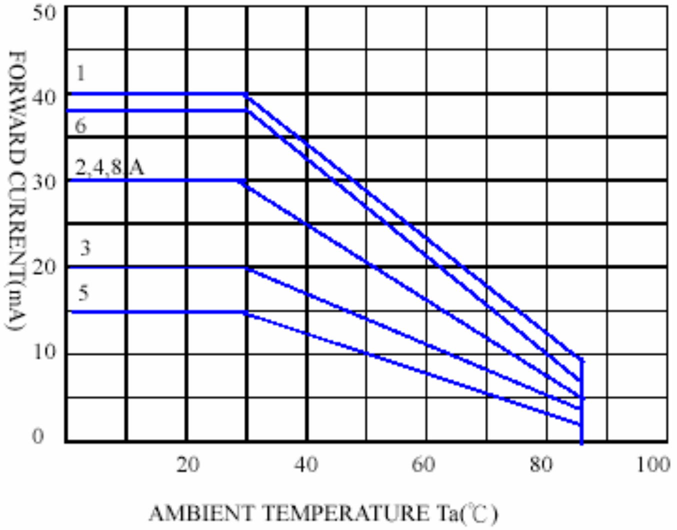

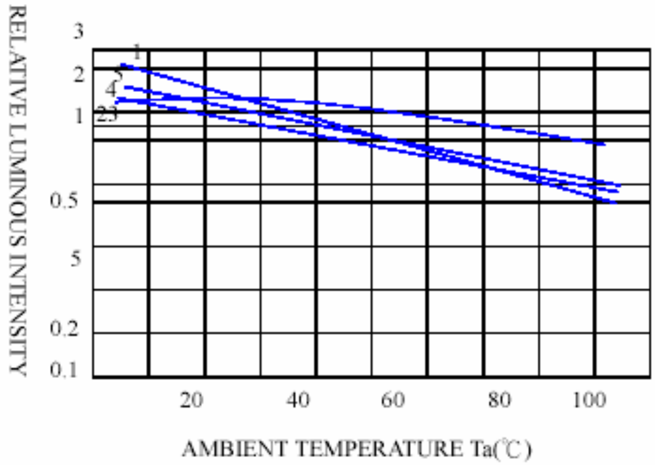

Supplement: Supplemental Information 2 [file peerj-07-6277-s002.pdf]
